# Nanoparticle delivery of a tetravalent E protein subunit vaccine induces balanced, type-specific neutralizing antibodies to each dengue virus serotype

**DOI:** 10.1371/journal.pntd.0006793

**Published:** 2018-09-24

**Authors:** Stefan W. Metz, Ashlie Thomas, Alex Brackbill, Yi Xianwen, Michele Stone, Katie Horvath, Michael J. Miley, Chris Luft, Joseph M. DeSimone, Shaomin Tian, Aravinda M. de Silva

**Affiliations:** 1 Department of Microbiology and Immunology, University of North Carolina, Chapel Hill, Chapel Hill, NC, United States of America; 2 Department of Pharmacology, University of North Carolina, Chapel Hill, Chapel Hill, NC, United States of America; 3 Lineberger Comprehensive Center, University of North Carolina, Chapel Hill, Chapel Hill, NC, United States of America; 4 Liquidia Technologies, Research Triangle Park, Durham, NC, United States of America; 5 Eshelman School of Pharmacy, University of North Carolina, Chapel Hill, Chapel Hill, NC, United States of America; 6 Department of Chemistry, University of North Carolina, Chapel Hill, Chapel Hill, NC, United States of America; University of Texas Medical Branch, UNITED STATES

## Abstract

Dengue virus (DENV) is the causative agent of dengue fever and dengue hemorrhagic shock syndrome. Dengue vaccine development is challenging because of the need to induce protection against four antigenically distinct DENV serotypes. Recent studies indicate that tetravalent DENV vaccines must induce balanced, serotype-specific neutralizing antibodies to achieve durable protective immunity against all 4 serotypes. With the leading live attenuated tetravalent DENV vaccines, it has been difficult to achieve balanced and type-specific responses to each serotype, most likely because of unbalanced replication of vaccine viral strains. Here we evaluate a tetravalent DENV protein subunit vaccine, based on recombinant envelope protein (rE) adsorbed to the surface of poly (lactic-co-glycolic acid) (PLGA) nanoparticles for immunogenicity in mice. In monovalent and tetravalent formulations, we show that particulate rE induced higher neutralizing antibody titers compared to the soluble rE antigen alone. Importantly, we show the trend that tetravalent rE adsorbed to nanoparticles stimulated a more balanced serotype specific antibody response to each DENV serotype compared to soluble antigens. Our results demonstrate that tetravalent DENV subunit vaccines displayed on nanoparticles have the potential to overcome unbalanced immunity observed for leading live-attenuated vaccine candidates.

## Author summary

Dengue virus (DENV) is the causative agent of dengue fever and dengue hemorrhagic fever. Yearly, over 350 million individuals in over 120 countries are infected. To establish protection through vaccination, one must induce simultaneous immunity against four antigenically distinct DENV serotypes. However, this is challenging because it has been shown that vaccination can enhance disease due to specific immunity to the virus. As an alternative to existing vaccine platforms, we are exploring the potential of a protein subunit vaccine using only the DENV envelope protein (E) as the vaccine antigen. To increase the immunogenic potency of E, we attach it to nanoparticle carriers. For each individual DENV serotype, we show that we can enhance immune responses in monovalent as well as tetravalent formulations when E is attached to nanoparticles. Additionally, in tetravalent nanoparticle formulations, vaccine quality is increased by the generation of a more balanced serotype specific immune antibody response to each DENV serotype. The nanoparticle vaccine platform described here for DENV vaccines serves as a promising and safe alternative to more conventional vaccine platforms and can be modified to develop vaccines for other viral pathogens such as West Nile, yellow fever virus or Zika virus.

## Introduction

The four dengue virus (DENV) serotypes are the causative agent of dengue fever and dengue hemorrhagic fever. DENVs are transmitted by *Aedes sp*. mosquitoes and both virus and vector are widely distributed throughout all tropical and subtropical regions, resulting in an estimated 300 million new infections per year, and approximately 1 million cases of severe disease with a case fatality 2–5% [1]. DENVs are endemic in over 125 countries and about 40% of the world’s population is at risk of getting infected by one of the 4 DENV serotypes. Primary infections induce robust and long term protective immunity against the serotype of infection, but individuals remain susceptible to one of the other serotypes. People experiencing secondary heterotypic infections are at greater risk of developing severe disease. Under some conditions, DENV serotype cross-reactive and poorly neutralizing antibodies induced after the primary infection, appear to enhance the second infection via the formation of virus-antibody complexes that promote infection of Fc-receptor bearing human myeloid cells [2,3]. It has been challenging to control the main mosquito vector of DENV. There are no effective antiviral or other therapies to treat DENV infections [4].

Based on success with other flaviviruses such as yellow fever and Japanese encephalitis viruses, vaccination is a promising strategy for dengue prevention and control. As effective immunity to just one serotype may place people at risk of severe disease upon infection with a different serotype, leading vaccine candidates are based on tetravalent live-attenuated virus formulations. In December 2015, the first DENV tetravalent vaccine, Dengvaxia developed by Sanofi Pasture, was licensed by several countries. However, long-term data from Dengvaxia clinical trials indicate that the vaccine is only effective in people who have already been primed by natural DENV infections before vaccination. Naïve individuals who have received the vaccine appear to face a greater risk of developing severe disease upon exposure to wild type DENVs and the vaccine is now recommended for use only in people with pre-existing immunity to DENVs [5–10].

As an alternative to inactivated or live attenuated whole virus formulations, several groups have focused on using recombinant DENV envelope (E) protein (rE) as a vaccine antigen [11–15]. Even though single soluble subunits are generally not immunogenic in primates [16], studies have shown that the immunogenicity of rE subunits can be augmented when combined with potent adjuvants or carriers [15,17,18]. We have previously demonstrated that adsorbing DENV2 rE to the surface of 80x320 nm particle replication in non-wetting templates (PRINT) produced poly(lactic-co-glycolic acid) (PLGA)-nanoparticles outperformed soluble rE subunits in terms of DENV2 specific IgG titers and neutralizing antibody titers [15]. The use of nanocarriers composed of biodegradable polymers not only creates an antigen depot and enhances antigen immunogenicity by effectively targeting antigen-presenting cells, but it provides a platform with the potential to mimic structural and antigenic features of the pathogen [19–22]. Here we demonstrate that a tetravalent DENV E protein formulation of PLGA nanoparticles induced higher levels of serotype specific IgG and neutralizing antibody titers than soluble tetravalent rE formulations in mice.

## Materials and methods

### Cells and viruses

Vero cells (American Type Culture Collection (ATCC)) were maintained as monolayer cultures in DMEM medium (Gibco) supplemented with 1% non-essential amino acids, 5% fetal bovine serum (FBS), 100 U/ml penicillin and 100 μg/ml streptomycin at 37°C with 5% CO_2_.

EXPI293 cells (Gibco) were maintained in suspension culture in EXPI293 Expression Medium (Life Technologies) and were passaged 1:10 when cell densities reached 3.5×10^6^ cells/ml.

To determine (neutralizing) antibody titers, DENV1 WestPac-74, DENV2 S-16803, DENV3 CH53489 and DENV4 TVP-376 were used.

### Recombinant E protein expression and purification

The recombinant E (rE) proteins of DENV1 (aa 1–397), DENV2 (aa 1–395), DENV3 (aa 1–395) and DENV4 (aa 1–397) were expressed by the EXPI293 transient expression system (ThermoFisher) following supplied protocols. The previously described expression construct was used [15] where an N-terminal IL2 secretion peptide leads the homotypic prM-rE cassette for each serotype. All rE proteins were equipped with a C-terminal 6×His-tag (SSGGSHHHHHH). Protein expression was driven by a CMV early enhancer β-actin promoter.

Recombinant proteins were purified as previously described [15]. In short, expression supernatants were concentrated and buffer exchanged by tangential flow filtration and proteins were purified by Ni^2+^-affinity chromatography. Elution fractions containing the rE-proteins were pooled and subjected to size-exclusion chromatography. Finally, the purified and concentrated proteins were flash frozen and stored at -80°C until further use.

### Protein analysis

Purified rE proteins were subjected to SDS-PAGE and analyzed by Western Blot (WB) and Coomassie Brilliant Blue (CBB) staining. 500 ng of DENV1 rE and 1μg of DENV2, 3 and 4 rE was added to a denaturing gel loading buffer containing SDS. Separated protein fraction were transferred to a nitrocellulose membrane and blocked overnight at 4°C with TBS+3% Skim Milk + 0.05% Tween-20. Next, the membranes were treated with 0.5 μg/ml 1M7 human mAb in blocking buffer for 1 hr at 37°C. After washing, the membranes were subjected to 1:1000 diluted AP-conjugated anti-human IgG for 1 hr at 37°C and membranes were subsequently washed. Finally, membranes were developed using NBT/BCIP substrate (ThermoScientific) and the reaction was terminated in MilliQ water.

### Protein confirmation analysis by antigen capture ELISA

Ni^2+^-coated ELISA plates (Pierce) were used to capture 2 ng/μl (in TBS) rE proteins (DENV1-4) for 1 hr at 37°C. The plates were blocked using TBS + 3% skim milk + 0.05% Tween-20 for 1 hr at 37°C and subsequently washed 3 times with TBS + 0.05% Tween-20. Next, plates were incubated for 1 hr at 37°C with 2 ng/μl (in blocking buffer) of mouse and human derived mAbs: 4G2 (mouse, cross-reactive), 3H5 (mouse, DENV2 specific), 12C1.5 (mouse, cross-reactive), 8A1 (mouse, DENV3 specific), 1M7 (human, cross-reactive), 1F4 (human, DENV1 specific), 2D22 (human, DENV2 specific), 5J7 (human, DENV3 specific), 5H2 (chimpanzee, DENV4 specific) and DV4 141 (human, DENV4 specific) (**Table 1**). Following incubation, the plates were washed and accordingly treated with AP-conjugated anti-mouse IgG (Sigma, 1:1000) or AP-conjugated anti-human IgG (Sigma, 1:2500) for 45 mins at 37°C. Finally, the plates were washed, developed with AP-substrate (Sigma) and absorbance was measures at 405 nm.

**Table 1:** Dengue specific monoclonal antibodies.

mAb	M/H/C	Binding	Neutralization *(W/M/S)*	Binding region	Binding DENV serotypes					Ref
					*DV1*	*DV2*	*DV3*	*DV4*	ZIKV	
***4G2***	M	F-CR	W	DII FL	++	++	+++	+++	+++	[23]
***1F4***	H	DV1	DV1:S	DI/DII hinge Q	+++	-	-	-	-	[24]
***12C1*.*5***	M	D-CR	DV:S	DIII	++	++	+++	++	-	[25]
***2D22***	H	DV2	DV2:S ZIKV:W	DII/DIII Q	-	++	-	-	-	[26,27]
***5J7***	H	DV3	DV3:S	DI/DII Q	-	-	+++	-	-	[28]
***5H2***	C	DV4	DV4:S	DI Q	-	-	-	+	-	[29]
***1M7***	H	F-CR	M	DII FL	+++	++	+++	+++	+++	[30]
***3H5***	M	DV2	DV2:S	DIII LR	-	+++	-	-	-	[31]
***8A1***	M	DV3	DV3:S	DIII	-	-	+++	-	-	[32]
***DV4 141***	H	DV4	None	DIII	-	-	-	+++	-	[33]

A panel of well-defined mouse, human or chimpanzee (M/H/C) derived Mabs were used in this study. Flavivirus cross reactive (F-CR), dengue cross reactive (D-CR), weakly, moderately or strong (W/M/S) neutralizing, E-domain I, II, III (DI, DII, DIII), fusion loop (FL), lateral ridge (LR), quaternary (Q).

### PLGA nanoparticle fabrication and formulation

PRINT technology was used to manufacture 80×320 nm poly (lactic co-glycolic acid) (PLGA, 50:50, 35 kDa, Lakeshore Biomaterials) particles as previously described [34,35]. In short, PLGA and DC-cholesterol (Avanti Polar Lipids) were dissolved in chloroform (9:1 w/w ratio) and casted into a thin film on a PET-sheet (KRS Plastics). The film was oriented in order to contact the molds and carefully heated. Next, the film was split and the mold content was transferred to a second PET-sheet by passing through a laminator. Then water with 0.1% polyvinyl alcohol (PVOH) was added to the PET-sheet to release the nanoparticles (NPs). The harvested particles were sterilized and concentrated by sterile filtration and tangential flow filtration.

To adsorb the rE proteins to NP surfaces, rE was incubated with PLGA NPs in a 1% rE/NP (w/w%) ratio for 15 mins at room temperature in 0.1% PVOH in water with 9.25% sucrose to establish 100% adsorption efficiency for all serotypes.

### Mouse immunizations

Female Balb/c mice were purchased from Jackson Laboratory and used at 6–12 weeks of age. For the monovalent formulations of every serotype, each mouse was immunized subcutaneously in the flank with 5 μg soluble rE (n = 5), 5 μg rE+500 μg Alum (n = 5), PBS (Vehicle n = 3) or 5 μg adsorbed to 500 μg PLGA-NPs (n = 5). DENV2 immunization were previously described, but were included to generate complete overview [15].

The tetravalent formulation were divided into 2 groups. The tetra rE group (TR) combined 5 μg soluble rE of each serotype (n = 5). The NP-tetra rE group (NTR) is composed of 500 μg NPs combined with a mix of the four rE serotypes (NP+rE^DENV1-4^ (5 μg each, n = 5)). The tetra NP-rE group (TNR) combines four individually adsorbed NP-rE formulations of each DENV serotype (NP-rE^DENV1^ + NP-rE^DENV2^ + NP-rE^DENV3^ + NP-rE^DENV4^ (500 μg NPs + 5 μg rE)). All groups in both the monovalent and tetravalent studies were immunized with the same antigen dose at day 0, 21 and 63 and serum samples were collected at indicated time points.

### Vaccine induced antibody evaluation

ELISA plates were coated overnight with 2 ng/μl 1M7 in 50mM carbonate/bicarbonate buffer at 4°C. The following day, the plates were blocked with TBS + 3% skim milk + 0.05% Tween-20 for 1 hr at 37°C. Next, the plates were washed in TBS + 0.05% Tween-20 and incubated with DENV2 or 2 ng/μl rE in blocking buffer for 1 hr at 37°C. After washing, the immunized mice sera of week 3, 4, 8, 10 and 16 were diluted 1:50 in blocking buffer and week 16 sera was also serially diluted in blocking buffer and loaded onto the plate for 1 hr at 37°C. Next, the plates were washed and incubated with AP-conjugated anti-mouse IgG (Sigma, 1:1000 in blocking buffer), IgG1 (Abcam, 1:2500) or IgG2a (Abcam, 1:2500) for 45 mins at 37°C. The plates were developed after washing with AP-substrate (Sigma) and absorbance was measured at 405 nm. The end point dilution (EPD) where the immunized mice sera reached background levels was determined using GraphPad Prism software.

### Evaluation of neutralizing antibody responses in immunized mice sera

The previously described Vero-cell based flow cytometry neutralization assay was used to measure DENV serotype specific neutralizing antibodies [15,36]. In brief, Vero cells (25000/well) were seeded and incubated overnight at 37°C. The next day, immunized mice sera was serially diluted in OptiMEM (Gibco) supplemented with 2% FBS and incubated with the appropriate amount of virus to infect ~15% of the cells (amount previously determined) for 45 mins at 37°C. Next, the cells were washed with OptiMEM and overlaid with the virus-serum combination for 2 hr at 37°C. Following incubation, the cells are washed with growth medium and incubated overnight in 200 μl growth medium at 37°C. Next, the cells are washed with PBS and detached from the plates by trypsin (Gibco). Detached cells are transferred to a round-bottom plate and fixed with 4% paraformaldehyde for 10 mins at room temperature. The cells are washed in permeabilization buffer and blocked 1% normal mouse serum in perm buffer for 30 mins at room temperature. Next, the cells were incubated with Alexa-fluor 488 conjugated anti-prM mAb 2H2 for 1 hr at 37°C. After washing in perm buffer, the cells were resuspended in 200 μl FACS buffer. The percentage of infected cells was determined by flow cytometry using the Guava Flow Cytometer (EMD Millipore) and the neutralizing capacity was determined by GraphPad Prism and expressed in neut_50_ values (the dilution where 50% of the virus was neutralized).

### IgG depletion of immunized mice sera by recombinant E proteins

Serum from 5 immunized mice was pooled for each tetravalent group and the serotype specific and cross-reactive IgG populations were depleted using immobilized homo- and heterotypic rE proteins. First, 1 mg of HisPur Ni-NTA magnetic beads (ThermoFisher) was washed and equilibrated using PBS + 10mM imidazole. Equilibrated beads were incubated with 40 μg of rE or MBP-His proteins (control depletions) for 30 mins at 37°C on a rotator. The beads were placed in a magnetic stand and were washed 3 times with washing buffer and finally divided over 2 tubes for two rounds of depletion. Next, 400 μl of 1:10 diluted (PBS) pooled sera was incubated with the chelated rE-beads for 1 hr at 37°C. The beads were placed in a magnetic stand and the sera was transferred for the second round of depletion for 1 hr at 37°C. The depleted serum was separated from the beads and stored at 4°C for subsequent IgG ELISAs and neutralization assays. The percentage of type specific neutralizing antibodies in **Table 2**was determined by dividing the type specific neut_50_ (CR depl.) by the control neut_50_ (Ctrl depl.).

**Table 2:** Particulated rE induced a balanced serotype specific tetravalent neutralizing antibody response in mice.

**TR** *(rE DV1-4)*					
***Serotype***	***Undepl*.** ***(Neut***_***50***_***)***	***Ctrl depl*. *(Neut***_***50***_***)***	***TS depl*. *(Neut***_***50***_***)***	***CR depl*. *(Neut***_***50***_***)***	***TS neut***
**DV1**	*2398*	*912*	*50*	*53*	***6*.*1%***
**DV2**	*7244*	*3715*	*50*	*1819*	***49*.*6%***
**DV3**	*10232*	*4466*	*50*	*1891*	***41*.*2%***
**DV4**	*1778*	*1513*	*50*	*630*	***43*.*1%***
**TNR** (*NP-DV1 + NP-DV2 + NP-DV3 + NP-DV4*)					
**Serotype**	**Undepl.** **(Neut**_**50**_**)**	**Ctrl depl. (Neut**_**50**_**)**	**TS depl. (Neut**_**50**_**)**	**CR depl. (Neut**_**50**_**)**	**TS neut**
**DV1**	1995	1174	50	257	***22*.*8%***
**DV2**	6918	2691	50	1000	***37*.*8%***
**DV3**	3090	2454	50	1318	***54*.*8%***
**DV4**	1258	562	50	501	***97*.*8%***

The proportions of serotype-specific and cross-reactive neutralizing antibody titers per DENV serotype in sera of mice immunized with tetravalent vaccine formulations were evaluated using a DENV serum depletion assay. Serum was incubated with magnetic beads coated with rE of DENV1, 2, 3 or 4 and serotype specific neutralizing IgG titers were determined against all 4 rE serotypes. The neutralizing capacity of undepleted (Undepl.), handling control sera (Ctrl depl.), serotype specific depleted (TS depl.) and cross-reactive depleted (CR depl.) was expressed as Neut_50_ values and used to determine the percentage of serotype specific neutralizing antibodies (TS neut).

### Ethics statement

All experiments involving mice were performed according to the animal use protocol (IACUC ID:17–047) approved by the University of North Carolina Animal Care and Use Committee. The animal care and use related to this work complied with federal regulations: the Public Health Service Policy on Humane Care and Use of Laboratory Animals, Animal Welfare Act, and followed the Guide for the Care and Use of Laboratory Animals.

## Results

### Expression and characterization of recombinant E protein (rE) protein from DENV1-4

**Fig 1:**
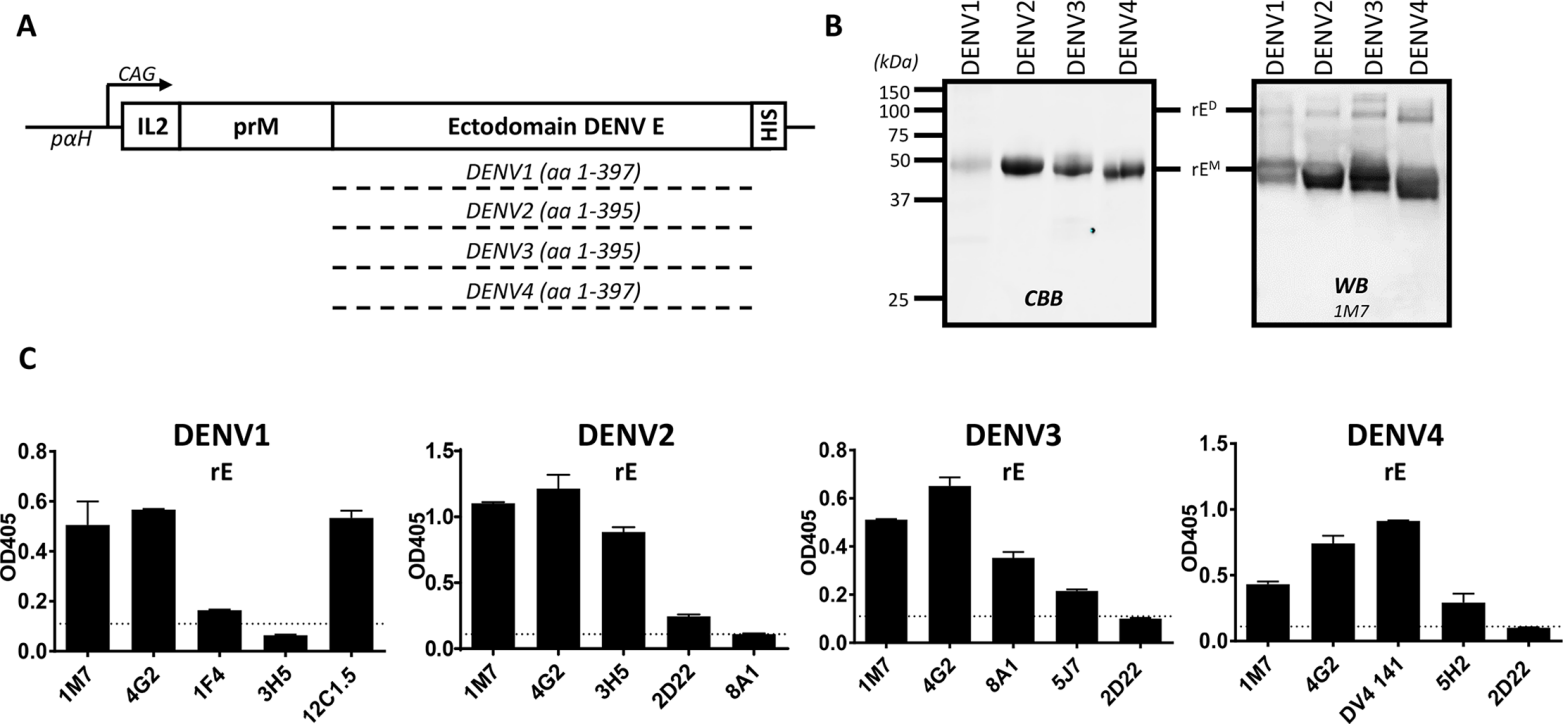
Expression and characterization of DENV1-4 rE. **A)** Schematic representation of the DENV1-4 rE expression constructs. The ectodomain of DENV1-4 E was expressed together with the homotypic prM protein, upstream an IL2 leader peptide. Recombinant proteins were equipped with a C-terminal His-tag and expressed under control of a CAG promoter on a p*α*H-expression vector in EXPI293 cells. **B)** Purified rE was subjected to SDS-PAGE and analyzed with CBB and WB using a 1M7 human derived mAb. **C)** DENV1-4 rE purified proteins fractions were loaded on Ni^2+^-coated ELISA plates and analyzed with a panel of cross-reactive or serotype specific mAbs (DENV1-1F4; DENV2-3H5, 2D22; DENV3-8A1, 5J7; DENV4-DV4 141, 5H2).

The full prM sequence and the ectodomain of E of each DENV serotype was cloned downstream of an IL2 secretion signal sequence and expressed in EXPI293 cells (**Fig 1A**). All rE proteins contained a C-terminal His-tag for purification and immobilization purposes. Analysis of the purified rE samples by gel electrophoresis and Western Blotting established the proteins were pure and of the predicted molecular mass of ~ 48kDa (**Fig 1B**). In addition to monomers, rE dimers of ~ 100kDa were routinely detected. While the exact confirmation of this dimer is unknown, DENV rE proteins have been shown to form a concentration and temperature dependent dimer-monomer equilibrium [37,38].

The DENV rE proteins were captured on Ni^2+^-coated ELISA plates using the C-terminal His-tag and tested for binding to a panel of serotype specific or cross-reactive mAbs (**Fig 1C**). The DENV cross-reactive mAbs 1M7 and 4G2 efficiently bound to all serotypes of rE. The DENV type-specific mAbs 3H5 (DENV2), 8A1 (DENV3), DV4 141 (DENV4) that recognize E protein epitopes on the monomer bound to each rE protein. DENV type-specific human mAbs 1F4 (DENV1), 2D22 (DENV2) and 5J7 (DENV3) which bind to quaternary structure epitopes displayed on the E homodimer or higher order structures showed marginal binding to each cognate antigen indicating that the purified antigens were mainly present as monomers [38].

### DENV rE antigens adsorbed to nanoparticles are better immunogens than soluble antigens

**Fig 2:**
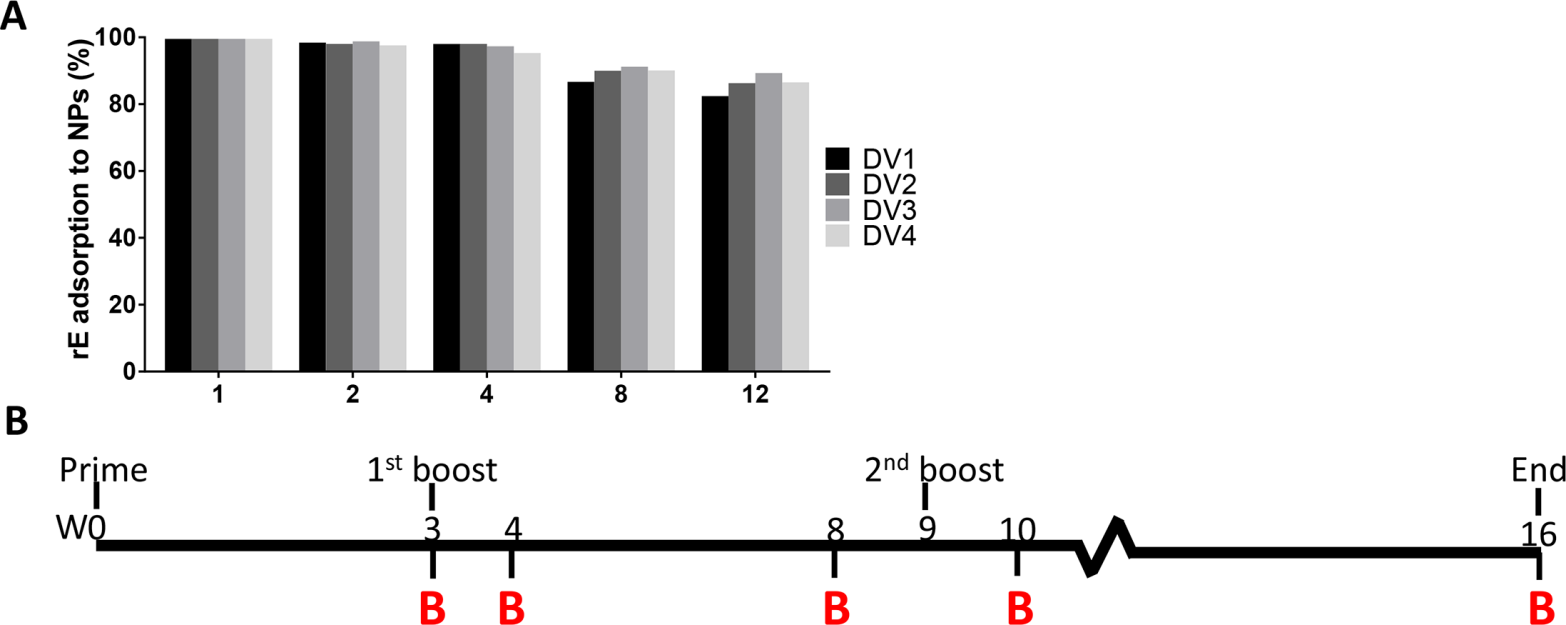
NP-rE adsorption efficiency. **A**) rE proteins from all serotypes were individually adsorbed to the PLGA nanoparticles in variable srE/NP (w/w%) ratios. Adsorption efficiency was determined by the amount of non-adsorbed rE after the spinning down the particles. **B)** Mice were immunized (subcutaneous) with 5 μg of rE or 5 μg rE adsorbed to PLGA nanoparticles. Animals were boosted on day 21 and day 63 and serum samples were taken at indicated time points.

**Fig 3:**
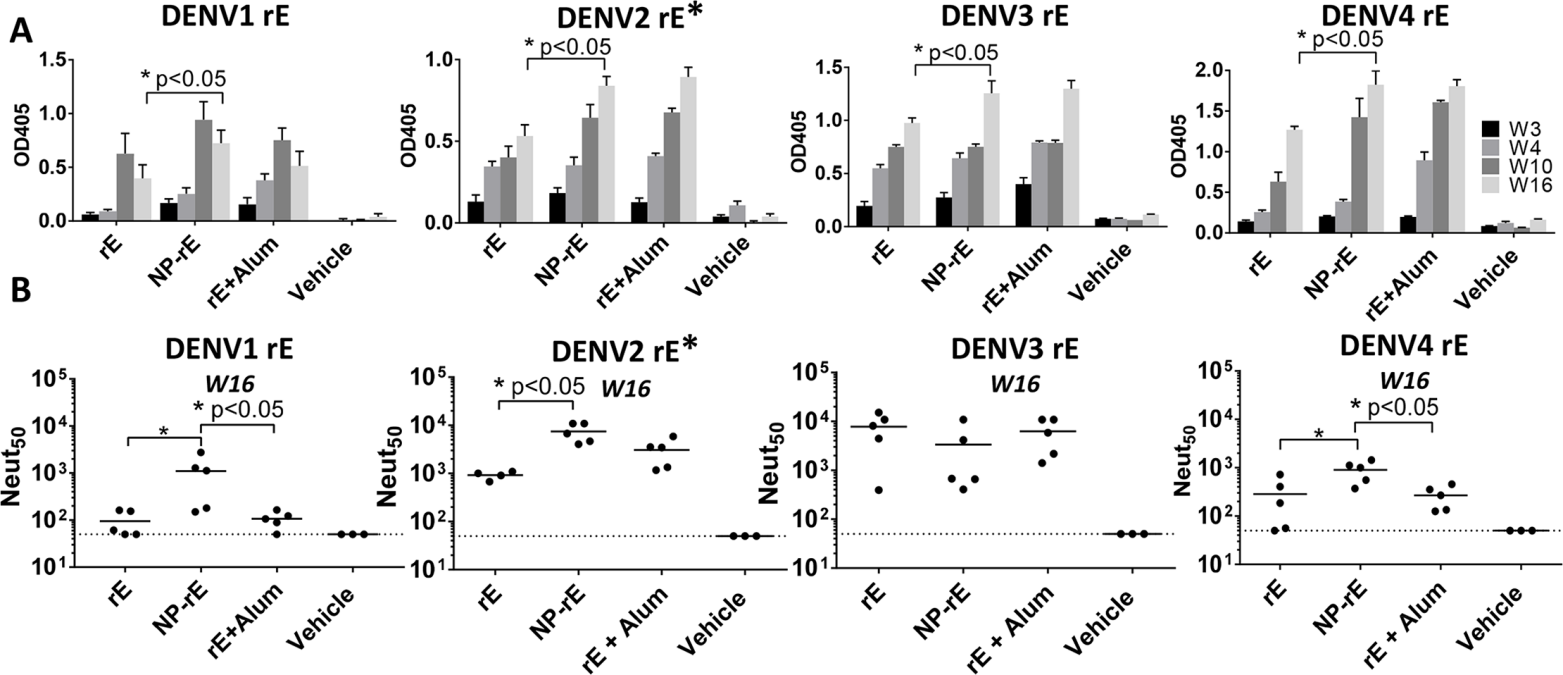
Monovalent NP-rE formulations induce robust neutralizing antibody titers. **A)** DENV1-4 specific IgG titers were determined for week 3, 4, 10 and 16. **B)** The neutralizing activity of the mice sera at week 16 was determined with a neutralization assay where DENV is incubated with serially diluted mice sera and subsequently allowed to infect Vero-cells. Neutralizing activity was expressed as the dilution where 50% of the virus was neutralized (Neut_50_). * DENV2 data adapted from previously published data [15]. Statistical differences were determined by one-way ANOVA followed by Tukeys test (p<0.05).

Mice were subcutaneously immunized with 5 μg of soluble rE alone, 5 μg of rE adsorbed to 80×320 PLGA NPs (**Fig 2A**) and 5 μg of rE with Alum (500 μg alum) on day 0 and then boosted on week 3 and week 9 with the same vaccine formulation used to prime the animals. DENV specific IgG levels were evaluated in sera collected on week 3, 4, 10 and 16, and neutralizing antibody titers were determined for week 16 serum samples (**Fig 2B**). The DENV1, 2 and 4 rE antigens adsorbed to nanoparticles stimulated higher mid-point dilution levels of specific antibody compared to soluble antigens (**Fig 3A**). The antigens on nanoparticles and the soluble antigens with Alum adjuvant induced similar levels of DENV-specific antibodies (**Fig 3A**). The DENV3 rE appeared to be inherently immunogenic, since DENV3 rE alone induced high levels of neutralizing antibodies that were not improved by particulation or the addition of the adjuvant. As described previously, at week 16 the DENV2 rE on nanoparticles induced higher levels of neutralizing antibodies than the soluble antigen alone (**Fig 3B**) [15]. Mice that were immunized with DENV1 or DENV4 rE on nanoparticles also had higher levels of neutralizing antibodies compared to soluble antigen alone or with the alum adjuvant (**Fig 3B**). These results establish that the immunogenicity of monovalent DENV1, 2 and 4 rE antigens is improved by adsorption to 80 x 320 nm PLGA nanoparticles when compared to the soluble antigen alone.

### The immunogenicity of tetravalent rE nanoparticle vaccines

**Fig 4:**
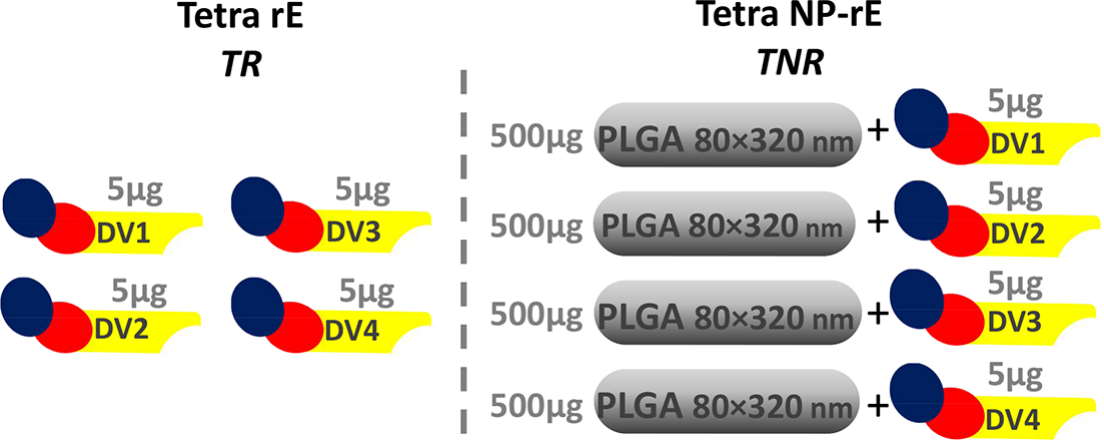
Tetravalent DENV1-4 rE formulations. The tetravalent formulations were divided into 2 groups. The tetra rE group (TR) combined 5 μg soluble rE of each serotype (n = 5). The tetra NP-rE group (TNR) combines four individually adsorbed NP-rE formulations of each DENV serotype (NP-rE^DENV1^ + NP-rE^DENV2^ + NP-rE^DENV3^ + NP-rE^DENV4^ (500 μg NPs + 5 μg rE).

**Fig 5:**
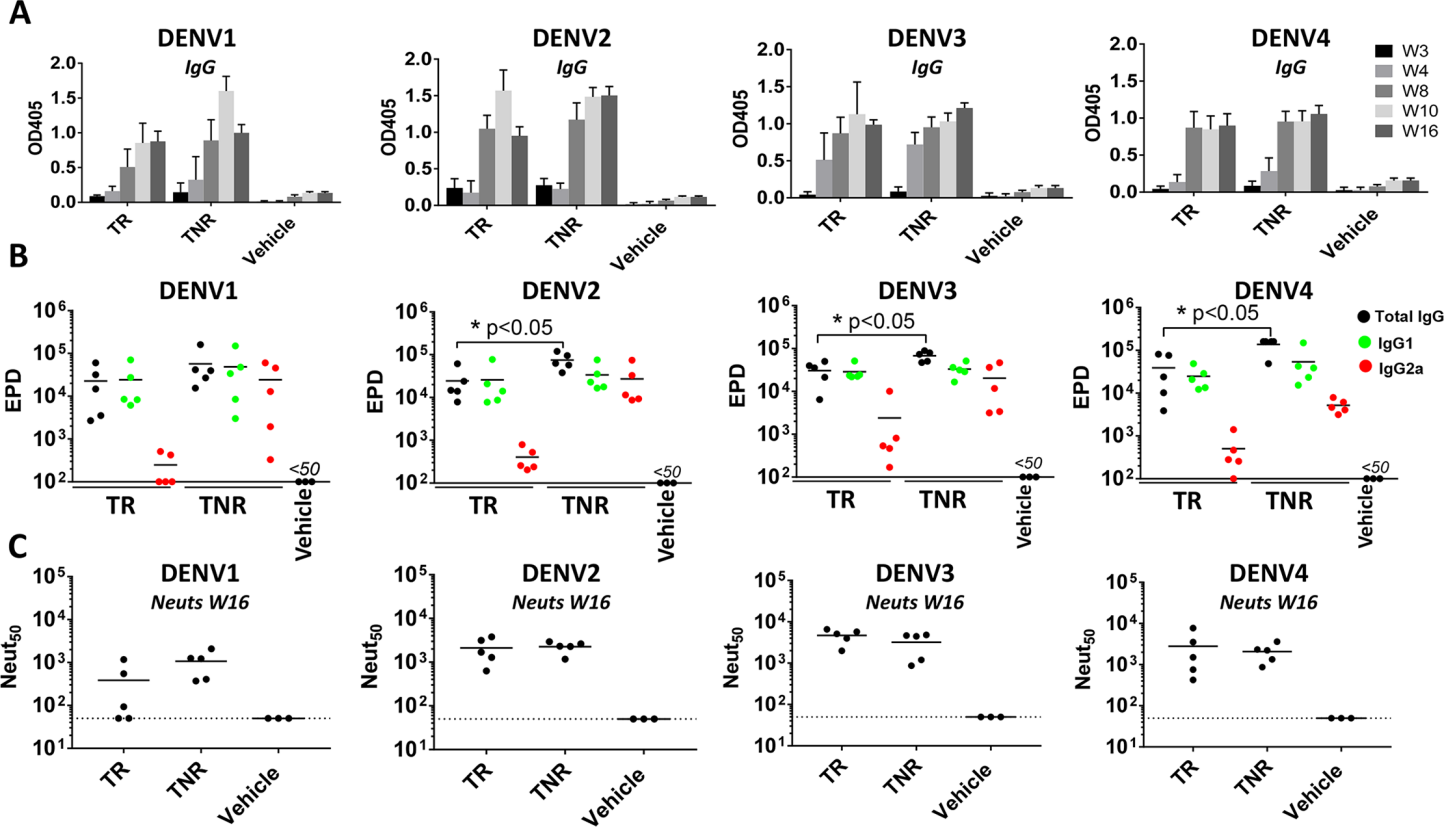
Tetravalent NP-rE immunization induces robust neutralizing IgG responses. **A)** DENV1-4 specific IgG responses were determined for week 3, 4, 8, 10 and 16. **B)** DENV1-4 specific IgG end-point dilution titers (EPD) for total IgG, IgG1 and IgG2a were determined for week 16 sera. **C)** The neutralizing activity of the mice sera at week 16 was determined with a neutralization assay where DENV is incubated with serially diluted mice sera and subsequently allowed to infect Vero-cells. Neutralizing activity was expressed as the dilution where 50% of the virus was neutralized (Neut_50_). Statistical differences were determined by one-way ANOVA followed by Tukeys test (p<0.05).

Next, we assessed the immunogenicity of tetravalent formulations of rE from DENV1-4 in the presence or absence of nanoparticles. Mice were immunized with a mixture of the 4 soluble rE antigens (5 μg per antigen) alone (TR group), or with 5 μg of each antigen separately adsorbed to 500 μg of PLGA NPs and then combined into a tetravalent mix (TNR group) (**Fig 4**). Mice were immunized and boosted at week 3 and 9. Serum samples were collected at week 3, 4, 8, 10 and 16 to measure total DENV-specific IgG and functionally neutralizing antibody (week 16 only) (**Fig 2A**). Both tetravalent formulations induced DENV-specific IgG against all four serotypes and antibody titers remained elevated through week 16 (7 weeks post 2^nd^ boost) (**Fig 5A**). At week 16, the overall level of DENV2, 3 and 4 binding antibodies were higher in the TNR group compared to the soluble TR group (**Fig 5B).** Soluble antigens predominantly induced an IgG1 response, where the particulated antigen stimulate a more balanced IgG1/IgG2a response (**Fig 5B**). The soluble and particulate formulation induced high levels of neutralizing antibodies with no significant differences between groups (**Fig 5C**). Overall DENV1 neut_50_ titers were lower compared to the other serotypes due to large variation within groups especially in the TR group.

### The influence of nanoparticles on the quality of DENV neutralizing antibodies induced by tetravalent rE vaccine

**Fig 6:**
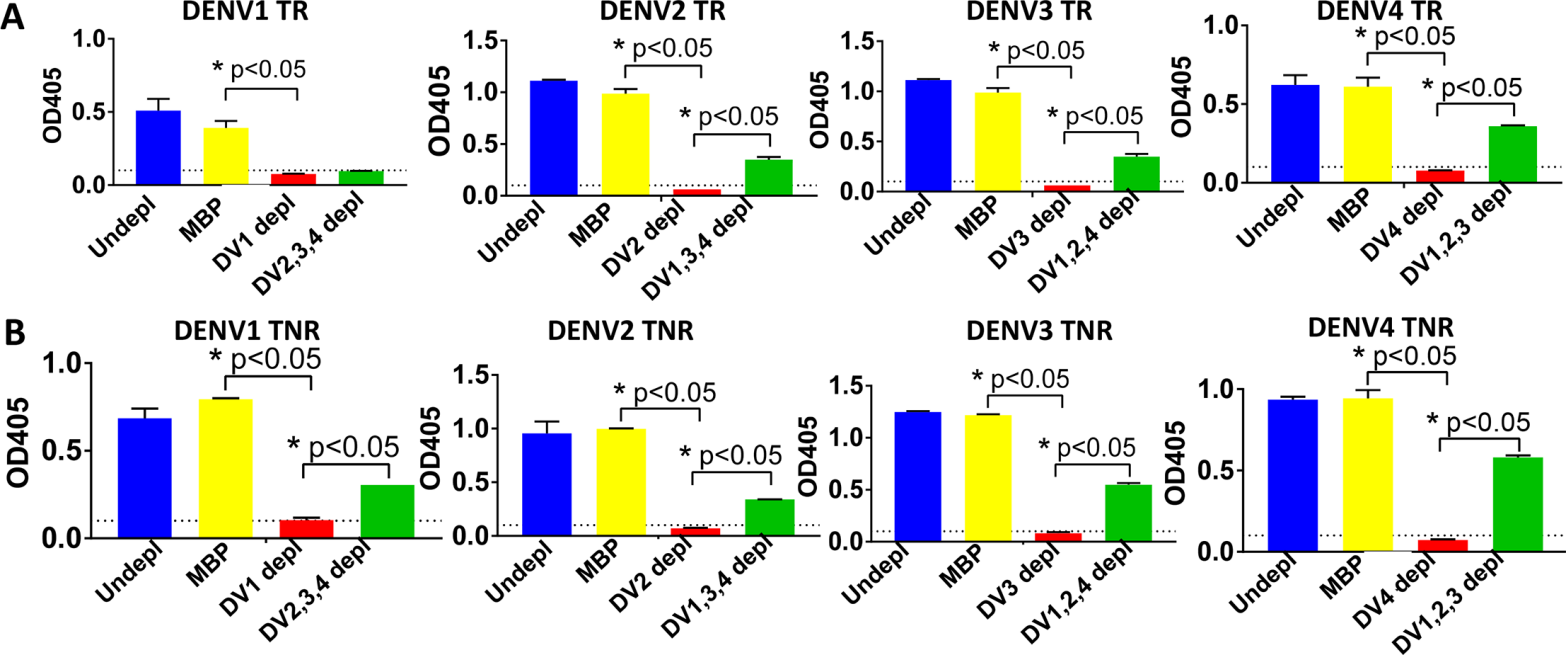
Tetra NP-rE induces serotype specific IgG responses in a tetravalent background. A DENV rE depletion assay was used to estimate the proportions of serotype-specific and cross-reactive antibody panels in sera of mice immunized with tetravalent vaccine formulations. Serum was incubated with magnetic beads coated with rE of DENV1, 2, 3 or 4 and serotype specific IgG titers were determined against all 4 rE serotypes for **A)** TR and **B)** TNR tetravalent formulations. For example, to establish complete exhaustion (red bars) of all serotype-specific and cross-reactive DENV1 binding antibodies, sera was depleted with DENV1 rE. To determine the DENV1 serotype-specific portion (green bars), the same sera was depleted with DENV2, 3 and 4 rE. A negative control or handling control depletion (yellow bars) was performed using maltose binding protein (MBP). Statistical differences were determined by one-way ANOVA followed by Tukeys test (p<0.05).

**Fig 7:**
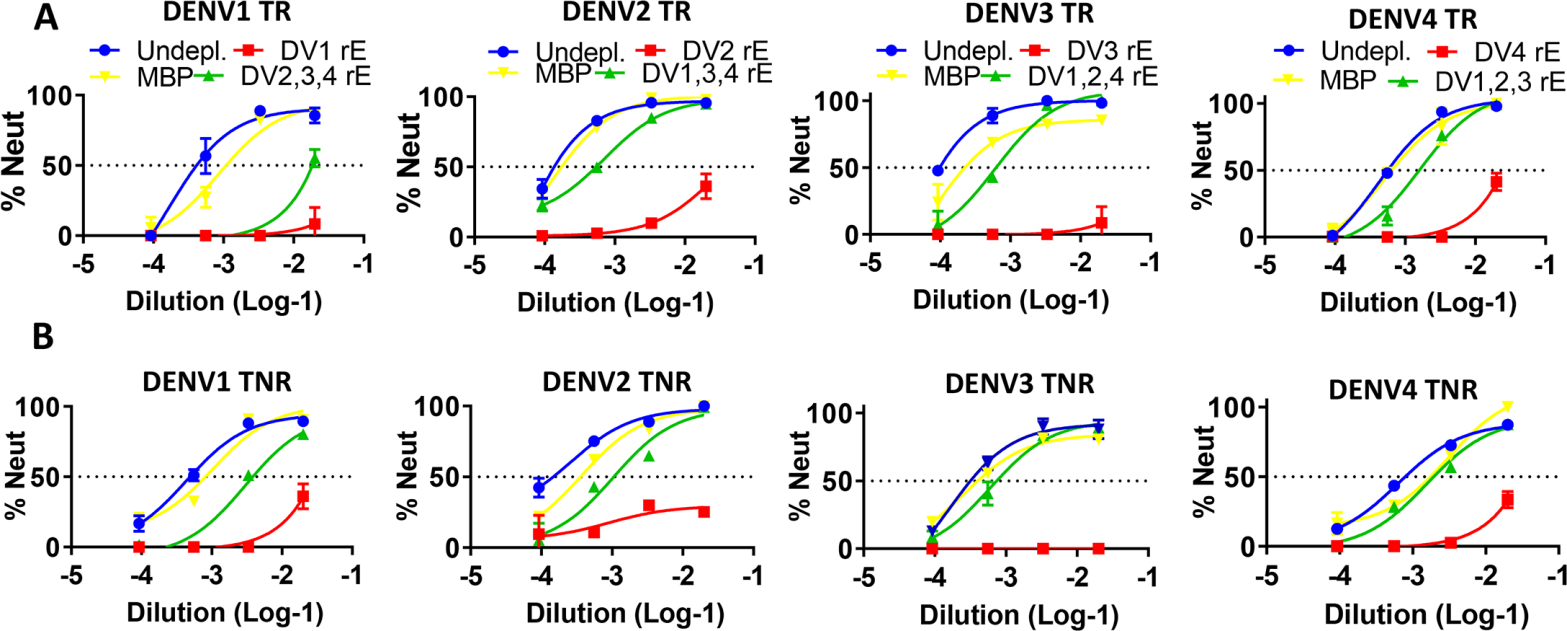
DENV rE particulation enhances serotype specificity in tetravalent formulations. To determine the portion of serotype-specific neutralizing antibodies, sera from **A)** TR and **B)** TNR immunized mice were depleted from serotype-specific and cross-reactive (red lines), cross-reactive (green lines) neutralizing antibodies. Serum neutralization was determined using a flow based approach where serially diluted serum was incubated with virus, which was subsequently allowed to infect vero-cells. Neutralizing capacity of the sera is expressed as Neut_50_ values, indicating the serum dilution where 50% of the virus is neutralized (**Table 1**).

Recent data from a live-attenuated DENV vaccine clinical trials indicate that the quality rather than the total quantity of DENV neutralizing antibodies is a better predictor of vaccine efficacy [39]. In particular, the level of DENV type-specific neutralizing antibody may be a better predictor of protective immunity than the total level of vaccine induced neutralizing antibody (type-specific + cross-reactive) [39]. We performed studies to evaluate the type-specificity of binding and neutralizing antibodies induced by soluble and nanoparticle delivered tetravalent rE vaccines. Given the limited quantities of week 16 immune sera remaining after the primary analysis, we had to pool the sera from the 5 mice in each group for these studies. To determine the fraction of serotype specific IgG and neutralizing antibodies stimulated by each formulation, the pooled immune sera from each group was depleted to remove total or just cross-reactive antibodies to each serotype and tested for binding to rE from each serotype (**Fig 6**) and for functional neutralization of each serotype (**Fig 7, Table 2**).

When DENV cross-reactive antibodies were depleted from immune sera collected from mice that received the soluble TR formulation, the animals had type-specific antibodies directed to epitopes on DENV2, 3 and 4 but not DENV1 (**Fig 6A green bars**). In the animals that received rE antigens adsorbed to nanoparticles, we observed type-specific antibodies to all 4 serotypes (**Fig 6B green bars**).

Depletions with the homologous rE of each serotype efficiently removed all neutralizing antibodies from mice immunized with each tetravalent formulation (**Fig 7, red lines**). In the TR group (soluble tetravalent antigen), the animals had high levels of type-specific neutralizing antibodies to DENV2, 3 and 4 but the DENV1 neutralizing antibody response was mainly derived from cross-reactive antibodies (**Fig 7A, green line; Table 1**). In contrast, in the TNR group the animals developed robust type-specific neutralizing antibody responses to all 4 serotypes (**Fig 7B, green line; Table 2**). The magnitude of the type-specific neutralizing antibody response was greater for both DENV4 and DENV1 in the TNR group compared to the TR group (**Table 2**). The DENV2 and 3 responses had similar levels of type-specific neutralizing antibodies in both the TR and TNR groups. Our results indicate that nanoparticle delivery of a tetravalent rE antigen mix stimulates a more balanced type-specific neutralizing antibodies compared to a soluble rE antigen mix.

## Discussion

A safe and efficacious vaccine that protects against all 4 DENV serotypes is urgently needed, but so far, results with leading live-attenuated tetravalent vaccine candidates are mixed. While the leading candidate developed by Sanofi Pasture is efficacious in people with pre-existing immunity to DENVs prior to vaccination, the vaccine is contraindicated in naïve individuals because of poor efficacy and safety [40]. The unbalanced replication of vaccine virus strains is the most likely explanation for the poor performance in naïve individuals [41]. Multi-component protein subunit vaccines are a promising alternative strategy to induce balanced immunity to all 4 serotypes but soluble antigens are poor immunogens compared to virions. Here we demonstrate that nanoparticles displaying rE from each serotype are a promising alternative to live virus vaccines.

In previous studies we have shown that adsorbing DENV2 rE to PLGA nanoparticles of 80 × 320 nm increased specific IgG and neutralizing antibody titers compared to the soluble rE protein alone [15]. In this study, we first evaluated nanoparticle delivery of the other three DENV serotypes in monovalent formulations. For DENV1, 2 and 4 we saw enhanced levels of serotype specific (TS) IgG and neutralizing antibodies when rE proteins were delivered using PLGA nanoparticles compared to soluble antigen. DENV3 rE appeared to be highly immunogenic as a soluble protein, so a particulation effect was not observed. Since all mice were inoculated with 5 μg of antigen, adsorbed to particles or as a soluble antigen, a lower DENV3 rE dose might reveal increased responses in the particle groups. This would require further dose optimization.

DENV1-4 rE proteins were evaluated as tetravalent soluble or particulate vaccines in mice. While both the soluble and particulate vaccines induced similar levels of neutralizing antibody, possibly due to an increase of cross-reactive antibody levels, we observed differences in IgG isotypes being induced. Where soluble antigens promote a predominant Th2 response by the induction of IgG1, particulate antigens stimulated a more balanced IgG1/IgG2a and thus Th1/Th2 response. In addition, we observed qualitative differences in the properties of neutralizing antibodies induced by each vaccine formulation. Though limited by the quantity of sera, serum depletion studies revealed a trend that the proportion of DENV serotype specific neutralizing antibodies increased when rE was adsorbed to PLGA surfaces compared to the soluble vaccine. This effect was especially clear for DENV1, and DENV4, where the serotype specific neutralizing antibody response against DENV1 increased from 6.1% to 22.8% and for DENV4 from 43.1% to almost 98%. Due large quantity of immune sera required for antibody depletion studies, we had to pool immune sera from all the animals within each group for these experiments, which precluded statistical analysis of the differences between groups.

While neutralizing antibodies have been long considered to be a surrogate of protective immunity in flavivirus vaccine development, the Sanofi clinical trial with Dengvaxia has clearly established that people with high levels of neutralizing antibodies can experience DENV infections [39,42]. The Sanofi experience and other studies point to both the level and quality of neutralizing antibodies as critical determinants of protective immunity [43,44]. In particular, in naïve individuals who are vaccinated, serotype-specific neutralizing antibodies appears to be critical for protection [39] [44]. Even though DENV rE subunit antigens have not been particularly promising as vaccine antigens [16], here we show that both the level and quality of neutralizing antibodies can be enhanced by using nanoparticles to deliver DENV monovalent and tetravalent rE proteins. The efficacy of subunit based dengue vaccines is expected be increased by the expression of quaternary epitopes recognized by strongly neutralizing antibodies. Although rE subunits are lacking these E-dimer dependent epitopes, we have previously developed methods to assemble E-dimers out of soluble monomers [37]. Future studies will focus on displaying quaternary epitopes carrying E-protein subunits on nanoparticles surfaces. In addition, our nanoparticles vaccine platform requires further evaluation of adjuvants to enhance the immunogenicity of particulated rE antigens. More broadly, a further developed platform described here for DENV vaccines can be modified to develop vaccines for other flaviviruses such as West Nile, yellow fever virus or Zika viruses.
